# Chronic urticaria: the first visit in a specialized unit

**DOI:** 10.1186/1939-4551-8-S1-A125

**Published:** 2015-04-08

**Authors:** Camila Teles Machado Pereira, Barbara M Aquino, Fernanda Cabral Cardoso Hardt, Luis Felipe Ensina, Inês Cristina Camelo Nunes, Dirceu Sole

**Affiliations:** 1 Federal University of Sao Paulo, Brazil; 2 Brazilian Society, Brazil

## Background

The aim of this study was to evaluate the clinical profile of patients with chronic urticaria (CU) at first visit in a specialized CU unit.

## Methods

Cross-sectional study of patients seen from January/2012 to June/2014. Patients diagnosed with acute urticaria (n=5) were excluded.

## Results

Among the 50 CU patients, 70% were female. The mean age at the consultation was 33 years, but the mean age of symptoms onset was 15.3 years. Fifty one percent referred only urticaria, 44% referred urticaria associated with angioedema and 5% presented isolated angioedema. Besides itching, 6 patients complained of burning (12%) and 2 of pain (4%). Frequency of wheals was daily in 33%, weekly in 36% and monthly in 31%. At first visit, Urticaria Activity Score was verified in 24 patients, resulting > 3 in half of them. The most associated atopic disease was rhinitis (45%). Many patients mentioned triggers as medicines (33%), food (23%), stress (17%), viral infection (8%) and physical agents (16%). Only 6% had thyroidopathy. As previous treatment a significant amount of patients received sedating antihistamines (AH) (36%) or oral corticosteroids (24%), with partial improvement in 65% and complete improvement in 29%. Dermographism was positive in 91% of the patients tested (20/22). One of them was diagnosed with cholinergic urticaria and another one with delayed pressure urticaria.

## Conclusions

The higher prevalence of CU was in middle-aged women. However, strong association with thyroiditis was not found. Sedating AH and corticosteroids are still the most prescribed drugs.

